# Pneumonia in Children

**Published:** 1850-07

**Authors:** 


*Pneumonia in Children.*—Dr. Mauthner, physician to the Vienna hospital for the diseases of children, has published a very sensible memoir on the management of a certain form of pneumonia in children. A valuable lesson is given by Dr. Mauthner on the subject of numeral statistics, and we call attention to this admonition, because it is in keeping with what we have often endeavored to impress on our readers. Dr. M. says:

"The numerical uniformity of mortality, as illustrated by the records of great hospitals in which pneumonia is treated in the most opposite ways, is exceedingly likely to deceive and mislead the young physician. I have lived long in hospitals, can speak from an eighteen years' experience, and know that when, in a great hospital, notwithstanding considerable occasional variations, the *average* mortality shows a fair proportion between the cures and deaths, one is usually apt to approve of the practice adopted in that hospital. Not so the *practical* physician. He regards each individual patient as an entire study; he cannot content himself with the reflection that a certain number of his cases of pneumonia are cured; he must seek to heal every case which occurs in his practice by every means at his disposal. The greatness of the practical man consists in *individualizing*, not in generalizing."

The pneumonia of children for which Dr. M. recommends venesection is charactized by the following symptoms:

"Difficult breathing, with oppression at the chest, short sharp cough (a symptom which, in the severe forms of the disease, is at first wanting,) heat of surface, fever, head affections, and sometimes vomiting.

"Over the seat of the disease, which is usually the back of the right lung, the percussion sound becomes dull; and, on ausculation in the early state, fine dry crepitation is audible; afterwards bronchial respiration. The child usually lies on the right side; and, if it can speak, complains of pain in the chest; the pulse is small, hard, and frequent; the secretions and excretions are diminished in quantity. These severe attacks are most common in children above a year old, but infants are not exempt from them."

The following sensible remarks are made by Dr. M. on the treatment:

"In the cases treated during the first stage, Mauthner has obtained the very best effects from blood-letting. He has often bled the little patients who have been brought for his advice to the hospital, permitted them to be carried home, and been subsequently astonished to find how completely this single remedy had obviated the urgent symptoms. On the other hand, he has too often seen the bad effects of neglecting this heroic remedy at the outset of the inflammation. In using the lancet, regard must be had to the age and constitution of the patient, and to the intensity of the disease. The depletion should not stop till the child turns pale, becomes sick, vomits, or seems exhausted. When the operation is properly performed, Mauthner thinks all other remedies, such as leeches, cataplasms, and internal physic, may be dispensed with, and in a few days the child is well.

"In cases in which the disease has reached its *second* stage, the immediate effect of venesection is not so remarkable; but some benefit is usually speedily experienced, and within three days the bronchial respiration ceases, and the morbid process is removed, usually upon the appearance of some critical evacuation from the skin or kidneys.

"Even when there was reason to suspect suppuration, Mauthner has often let blood after mature deliberation, and has had no occasion to repent the adoption of the practice. Thus, he believes that he succeeded in saving a boy six years of age, who, after a neglected pneumonia of three weeks' standing, fell under his care, suffering from hectic fever, emaciation, and purulent expectoration. After in vain attempting a cure with digitalis and acetat. plumbi, Mauthner ordered venesection, and the boy recovered. Where, however, as is often the case, an unresolved pneumonia seems to have gone on to tuberculosis, bleeding can do no good.

"The indications for venesection are derived, *first*, from an accurate physical exploration of the chest. It must, however, be noted, that, when the cough and general symptoms indicate the existence of pneumonia, the absence of physical signs does not justify the neglect of proper remedies. For, even when extensive congestion of the pulmonary tissue is present, if a certain quantity of air be still included in its interstices, the percussion sound and respiratory murmur may not be sensibly affected. Besides, the inflammation may be situated in a part of the lung which cannot be satisfactorily explored; or the child may be so restless and timid as to render the use of the stethoscope impossible.

The *second* indication is derived from the age and individual constitution of the child. Strong plump children above a year old may be bled without scruple; those who are delicate will still bear depletion, if it be ascertained that their previous health has been good; even infants under one year of age, laboring under severe pneumonia, suffer less from venesection than from the application of three or four leeches.

"The stage of the disease affords the *third* indication. In the congestive period, the remedy is quick and sure. When hepatization has already taken place, the detraction of blood is never hurtful; but, when the suppurative period has arrived, the lancet must be used cautiously and seldom.

"The operation of opening a vein in the arm of a fat restless child is not easy, and for its proper performance requires some previous experience, for the *feel* of the vein must, more than the eye, guide the lancet. The ribbon must at first be drawn rather tightly, until the vein be felt and opened, when it must immediately be slackened again. While the blood trickles out (a full jet is seldom obtained from a child,) the arm should be left quite still, for, on the slightest movement, the fat mobile integuments lap over and close the orifice. There is no difficulty in stopping the bleeding, either with a cross of sticking plaster, or by a single turn of a bandage.

"The amount of blood drawn must be chiefly regulated by the age of the child. Considerable effect may be expected in infants from the detraction of a single ounce. Children from two to three years of age require a blood-letting of from two to three ounces, and older children more in proportion."

